# Widespread distribution of *Plasmodium vivax* malaria in Mauritania on the interface of the Maghreb and West Africa

**DOI:** 10.1186/s12936-016-1118-8

**Published:** 2016-02-09

**Authors:** Hampâté Ba, Craig W. Duffy, Ambroise D. Ahouidi, Yacine Boubou Deh, Mamadou Yero Diallo, Abderahmane Tandia, David J. Conway

**Affiliations:** Institut National de Recherches en Santé Publique (INRSP), Nouakchott, Mauritania; Department of Pathogen Molecular Biology, London School of Hygiene and Tropical Medicine, London, UK; Hôpital Le Dantec, Université Cheikh Anta Diop, Dakar, Senegal

**Keywords:** *Plasmodium vivax*, Africa, Malaria, Endemic, Duffy positive

## Abstract

**Background:**

*Plasmodium vivax* is very rarely seen in West Africa, although specific detection methods are not widely applied in the region, and it is now considered to be absent from North Africa. However, this parasite species has recently been reported to account for most malaria cases in Nouakchott, the capital of Mauritania, which is a large country at the interface of sub-Saharan West Africa and the Maghreb region in northwest Africa.

**Methods:**

To determine the distribution of malaria parasite species throughout Mauritania, malaria cases were sampled in 2012 and 2013 from health facilities in 12 different areas. These sampling sites were located in eight major administrative regions of the country, within different parts of the Sahara and Sahel zones. Blood spots from finger-prick samples of malaria cases were processed to identify parasite DNA by species-specific PCR.

**Results:**

Out of 472 malaria cases examined, 163 (34.5 %) had *P. vivax* alone, 296 (62.7 %) *Plasmodium falciparum* alone, and 13 (2.8 %) had mixed *P. falciparum* and *P. vivax* infection. All cases were negative for *Plasmodium malariae* and *Plasmodium ovale*. The parasite species distribution showed a broad spectrum, *P. vivax* being detected at six of the different sites, in five of the country’s major administrative regions (Tiris Zemmour, Tagant, Brakna, Assaba, and the capital Nouakchott). Most cases in Nouakchott were due to *P. vivax*, although proportions vary significantly among different health facilities in the city. In the northern town of Zouérat, all cases were due to *P. vivax*, whereas almost all cases in the south of the country were due to *P. falciparum*. All *P. vivax* cases tested were Duffy blood group positive.

**Conclusions:**

It is important that *P. vivax* is recognized to be a widespread cause of malaria in Mauritania, occurring in diverse regions. This should be noted by the World Health Organization, as it has significant implications for diagnosis, treatment and control of malaria in the northwestern part of Africa.

## Background

Most malaria cases in Africa are caused by *Plasmodium falciparum* [1], with a relatively small proportion attributable to *Plasmodium malariae* or *Plasmodium ovale*, while *Plasmodium vivax* is known to occur in only some areas of the continent [2, 3]. Following scale-up of prevention and treatment, the incidence of malaria has significantly declined in some areas, contributing to a reduction in overall mortality [4, 5]. Although most of West Africa still has a high malaria burden, numbers of cases have markedly declined near the northwestern end of the endemic distribution [1, 6, 7]. More studies are needed in areas closer to the edge of endemicity, where seasonal transmission is normally very limited.

The country of Mauritania is at the extreme northwestern limit of malaria distribution in Africa. It has a population of approximately 3.5 million people, and covers a large area of 1,030,700 sq km^2^, lying between 15 and 27°N latitude, and 5 and 17°W longitude. Malaria is considered to be the third most common cause of clinical presentation to health facilities in the country. Although a large part of the territory is Saharan desert, malaria is most common in areas of Sahel in the south of the country, and has been increasingly seen in Nouakchott, the capital city that contains approximately 25 % of the country’s inhabitants [8–10]. Recent reports indicate that *P. vivax* is more commonly seen than *P. falciparum* in patients with malaria in Nouakchott [9–12]. This represents an apparently unique epidemiological situation, as *P. vivax* has been very rarely or never seen at most other sites in West Africa [2, 3, 13–15]. One factor contributing to this is that most inhabitants in Nouakchott are ethnically identified as white Maures, who are generally positive for the Duffy antigen erythrocyte receptor for *P. vivax*, which is rare among other West African ethnic groups living mostly in the south of the country [16]. A second factor is that local breeding of the mosquito vector *Anopheles arabiensis* has been facilitated by surface water environments, created by loosely regulated urbanization over recent decades in an area that was originally desert [12].

Outside of the capital Nouackchott, the limited slide microscopy surveys conducted historically have indicated that almost all malaria cases are due to *P. falciparum*, apart from occasional cases reportedly being due to *P. malariae* and *P. ovale* [8]. *P. vivax* has not been clearly described at other sites, except for a few infections detected in 2009 and 2010 in the Hodh El Gharbi region in the south of the country [17], although it has been suspected elsewhere. As there have been few recent surveys, and identification of species by slide microscopy is often unreliable, a broad survey using molecular methods for species identification is needed. In this study, the distribution of parasite species in malaria cases was surveyed by sampling from health facilities at diverse sites throughout Mauritania.

## Methods

**Fig. 1:**
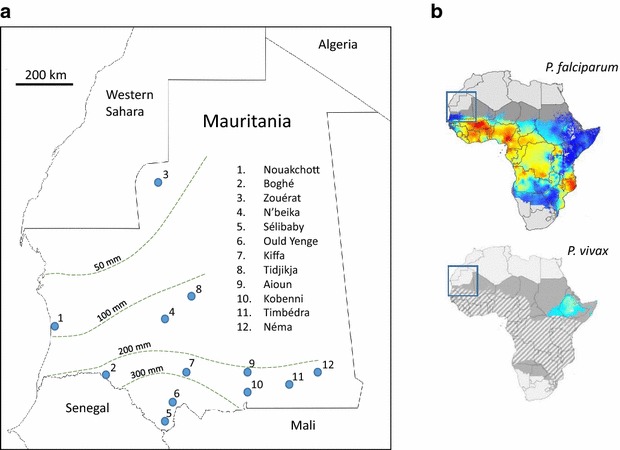
Map of Mauritania showing the locations of the sampling sites in relation to average annual rainfall and previously known areas of malaria endemicity. **a** Malaria patients were sampled from 12 locations in Mauritania. In the capital city Nouakchott (Location 1) sampling was conducted from three hospitals: Health Centre of Teyarett, Cheikh Zayed Hospital Center, and the National Hospital Centre. Sampling was conducted from single facilities in each of the other 11 locations: hospitals in Sélibaby, Aioun, Néma and Zouérat, and health centres in each of the other locations. Malaria parasite species were determined from 472 patients in total (numbers from each location are given in Table 1). The approximate mean annual rainfall in millilitres in different areas is indicated by isohyets shown as dashed lines. **b**. *Inset rectangles* indicate the position of the study area map on previously published maps of malaria parasite distribution in Africa [1, 3]. Estimated endemic distributions of *P. falciparum* and *P. vivax* in 2010 are indicated by coloured shading (*blue* indicating low prevalence, *yellow* and *red* indicating higher prevalences). *Grey* shading indicates where sporadic transmission is considered possible without endemic maintenance. In the lower map the diagonal shading indicates where the majority of the population has the Duffy-negative blood group phenotype

Patients with malaria were sampled from among those attending 14 health facilities at 12 different geographical sites in eight administrative regions of Mauritania in 2012 and 2013 (Fig. 1). Three of the facilities were within the capital city Nouakchott: the National Hospital Centre, Cheikh Zayed Hospital Centre and Teyarett Health Centre, the first of which serves a broad population of mixed ethnicity, while the other two serve areas predominantly inhabited by white Maures. The large urban area of Nouakchott typically receives only 50 to 100 mm annual rainfall but has a large supply of piped water from the south of the country. One of the other sites sampled was far outside of the known malaria-endemic area, in the mining town of Zouérat (Tiris Zemmour Region) in the north of the country, which usually receives less than 50 mm of rain annually. The remaining nine health facilities were in different towns and villages throughout ecologically diverse parts of the country known to be endemic for malaria: Boghé (Brakna Region) in the Senegal River valley generally receives between 200 and 300 mm annual rainfall; Sélibaby and Ould Yenge (Guidimakha Region) within 100 km of the Senegal River valley receive between 300 and 600 mm annual rainfall; Aioun and Kobenni (Hodh El Garbi Region), Timbédra and Néma (Hodh El Chargi Region), and Kiffa (Assaba Region) are in a semi-arid zone where average rainfall varies between approximately 100 and 300 mm; N’beika and Tidjikja (Tagant Region), are in an arid zone which usually receives only between 50 and 200 mm of rain annually (Fig. 1). The limited and variable annual rainfall in Mauritania mostly occurs between July and October, leading to seasonal malaria, so cases were sampled between August and December in each year.

Patients were diagnosed with uncomplicated clinical malaria by local health facility staff on the basis of clinical signs including the presence of fever, supported by positive rapid diagnostic tests detecting circulating malaria parasite lactate dehydrogenase (OPTIMAL-IT ^TM^ kits incorporating two different antibodies that are, respectively, *P. falciparum* species-specific and broadly *Plasmodium*-specific). Tests that are negative for *P. falciparum* but positive for *Plasmodium* are routinely considered to be likely due to either *P. malariae*, *P. ovale*, or *P. vivax*, although resolution of these alternative species is not done at diagnosis in most health facilities. All cases analysed were local residents who did not report having travelled within the previous 2 weeks, and who were invited to provide finger-prick blood samples. These were collected on filter paper and air-dried prior to storage with desiccant in sealed polythene bags. All samples were obtained with informed consent from patients, and guardians of patients who were under 18 years of age. Regardless of inclusion in the study, patients were treated at the health facilities with artemisinin combination therapy. Current recommendations of the National Malaria Control Programme are for first-line treatment combination of artesunate and amodiaquine, and second-line treatment option of artemether and lumefantrine, with primaquine being added in the case of confirmed *P. vivax* infection. The study was approved by ethics committees of the Ministry of Health of Mauritania and the London School of Hygiene and Tropical Medicine.

DNA was extracted from filter paper blood spots using QIAmp DNA Minikits (Qiagen, Valencia, CA, USA). Species-specific identification of malaria parasites by nested PCR was performed using a published method [18], with a first round of *Plasmodium* genus-specific PCR followed by species-specific amplification using primers to detect *P. falciparum*, *P. vivax*, *P. malariae*, and *P. ovale*. Products were separated electrophoretically on 1 % agarose gels, stained with ethidium bromide and visualized using ultraviolet trans-illumination for photography and scoring. Positive and negative controls were included in all assays, with DNA from different malaria species kindly provided by Colin Sutherland from the UK Malaria Reference Laboratory.

A proportion of individuals sampled were genotyped to test for Duffy blood group negativity, based on the GATA-1 gene promoter allele that leads to null expression in West African populations, using a previously published protocol [19].

## Results

**Fig. 2:**
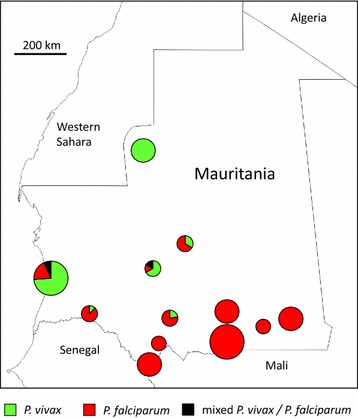
Proportions of *Plasmodium vivax* and *Plasmodium falciparum* malaria cases at each of 12 locations sampled in Mauritania shown by pie diagrams. The locations are as specified in Fig. 1. The size of pies varies according to the number of PCR positive cases analysed at each site: small, <20; medium, 20–100; large, >100. Exact numbers are given in Table 1

Overall, 472 patients sampled had malaria parasites detected by *Plasmodium* genus-specific as well as species-specific PCR analyses. Of these, 296 (62.7 %) had *P. falciparum* alone, 163 (34.5 %) *P. vivax* alone, and 13 (2.8 %) had mixed *P. falciparum* and *P. vivax* infections. None of the samples was positive for *P. ovale* or *P. malariae*. Apart from Nouakchott, in which infections were already known to occur, *P. vivax* is described here for the first time from five widely separated sites in four other administrative regions of Mauritania (Tiris Zemmour, Tagant, Brakna, and Assaba). The proportions of malaria cases with *P. vivax* and *P. falciparum* showed extreme variation among the different sites (Table 1). This ranged from a situation at six sites in the southeast of the country at which all cases detected were *P. falciparum*, through to the opposite extreme at Zouérat in the north of the country at which all cases were *P. vivax*. Both malaria parasite species were present at the remaining sites (Fig. 2).

**Table 1:** Proportions of *P. vivax* and *P. falciparum* infections detected in malaria cases sampled from 12 different areas in Mauritania

Location and year of sampling	Number positive	*P. vivax* alone	*P. falciparum* alone	*P. vivax* and *P. falciparum* mixed	Proportion of *P. vivax* *(Pv/[Pv* + *Pf])* ^a^
2012					
Aioun	30	0	30 (100 %)	0	0.00
Boghé	8	1 (13 %)	7 (88 %)	0	0.13
Kobenni	131	0	131 (100 %)	0	0.00
N’beika	6	4 (67 %)	1 (17 %)	1 (17 %)	0.71
Tidjikja	3	1	2	0	0.33
Timbédra	17	0	17 (100 %)	0	0.00
Nouakchott	96	65 (68 %)	20 (21 %)	11 (11 %)	0.73
2013					
Sélibaby	29	0	29 (100 %)	0	0.00
Kiffa	9	2 (22 %)	7 (78 %)	0	0.22
Ould Yenge	14	0	14 (100 %)	0	0.00
Zouérat	25	25 (100 %)	0	0	1.00
Néma	24	0	24 (100 %)	0	0.00
Nouakchott	80	65 (81 %)	14 (18 %)	1 (1 %)	0.81

Individual locations are shown on Fig. 1. Samples from Nouakchott were from three different health facilities, and proportions of species at each of these are shown in Table 2

^a^Relative proportions of each species at each site are estimated by counting numbers of infections with each species, with each being counted separately from mixed species infections

In the capital Nouakchott, *P. vivax* was more common than *P. falciparum* in both 2012 and 2013. Overall, there was no significant difference between the 2 years in the relative proportion of the two parasite species. However, within each year there were differences in the proportions of species between the different health facilities sampled in Nouakchott (Table 2). At Teyarett Health Centre in both years, most malaria cases were caused by *P. vivax*, whereas in the other two hospitals sampled separately in the respective years there were more equal proportions of both *P. vivax* and *P. falciparum*. In 2012, 85 % (49/53) of cases sampled from Teyarett Health Centre had *P. vivax* alone, in contrast with 37 % (16/43) at the National Hospital Centre (Fisher’s Exact P < 0.0001). In 2013, 85 % (62/73) of cases sampled from Teyarett Health Centre had *P. vivax* alone, in contrast with 43 % (3/7) at the Cheikh Zayed Hospital Centre (Fisher’s Exact P = 0.013).

**Table 2:** Proportions of malaria infections containing *P. vivax* and *P. falciparum* at each of three health facilities sampled in Nouakchott, the capital of Mauritania

Health facility and year of sampling	Number of cases sampled	*P. vivax* alone	*P. falciparum* alone	*P. vivax* and *P. falciparum* mixed	Proportion of *P. vivax (Pv/[Pv* + *Pf])* ^a^
2012					
Teyarett Health Centre	53	49 (92 %)	4 (8 %)	0	0.92
National Hospital Centre	43	16 (37 %)	16 (37 %)	11 (26 %)	0.50
2013					
Teyarett Health Centre	73	62 (85 %)	10 (14 %)	1 (1 %)	0.84
Cheikh Zayed Hospital Centre	7	3 (43 %)	4 (57 %)	0	0.43

^a^Relative proportions of each species at each hospital are estimated by counting numbers of infections with each species, with each being counted separately from mixed species infections

Duffy promoter genotyping of 82 *P. vivax* positive cases did not show any individual to be homozygous Duffy negative.

## Discussion

In contrast to the situation in other West African countries, *P. vivax* is a widely distributed cause of clinical malaria in Mauritania. Cases occur over a large area, and are not restricted to the capital Nouakchott from where they were previously described. Remarkably, there is no information on the occurrence of this parasite species in country malaria reports for Mauritania given by the World Health Organization [5]. This study shows that the species exists alongside *P. falciparum* in the central and southwest parts of the country (Tagant, Brakna and Assaba regions), and in Nouakchott where its relative frequency as a cause of malaria varies between different health facilities. Notably, *P. vivax* was the only parasite species seen in malaria patients from Zouérat in the north of the country (Tiris Zemmour region), whereas in the southeast of the country it is clear that *P. falciparum* dominates and *P. vivax* is rarely seen.

The epidemiology of malaria in these communities needs to be better understood. It is possible that *P. vivax* might be maintained in areas with minimal transmission in the centre and north of the country due to long persistence of infection in individuals experiencing occasional relapse from dormant hypnozoite stages in the liver. As samples were from individuals who had not reported travelling within the previous 2 weeks, most of the cases were probably acquired locally, although some might have been due to relapses of *P. vivax* infections acquired previously. A study of malaria in northern Senegal near the border with Mauritania surveyed households of index cases [20], revealing that many *P. falciparum* infections are associated with travel to more highly endemic parts of Senegal. Surveys based around index cases may also be useful to understand the epidemiology of *P. vivax* as well as *P. falciparum* in different parts of Mauritania. Although there is a previous report of a *P. vivax*-infected individual in Nouakchott who was Duffy negative [16], in the present study all cases of *P. vivax* tested were shown to be Duffy positive. Indeed, where *P. vivax* was seen to be clearly the predominant cause of malaria, for example in the Teyarett area of Nouakchott and in the northern town of Zouérat, most of the local population are white Maures, an ethnicity highly associated with Duffy positivity. Therefore, this parasite species will probably remain rare in the extreme south of Mauritania where most of the population are Duffy negative, but its widespread distribution in the central and northern parts of the country needs to be monitored and addressed.

Although the distributions of *P. vivax* and *P. falciparum* differ globally [21], it is remarkable that *P. vivax* as a widespread cause of malaria in communities living in desert and Sahel areas of West Africa has not been previously appreciated. The situation described here in Mauritania, and recent data on a limited number of positive samples in Senegal [22] and northern Mali [14, 15], suggest that *P. vivax* is likely to be endemic in many communities living in other parts of northwestern Africa which have yet to be surveyed with species-specific methods [2]. There is recent evidence suggesting that *P. vivax* emerged prehistorically as a zoonosis from African apes prior to becoming endemic in humans and then spreading to other continents [23]. It remains to be determined whether the current *P. vivax* distribution in Mauritania is part of a relict African parasite population, or whether the species was re-introduced in historical times.

## Conclusions

*Plasmodium vivax* causes a substantial proportion of malaria cases in diverse parts of Mauritania. Far from being restricted to the capital Nouakchott, *P. vivax* is detected from five of the major administrative regions of this large country, including one northern town in which all cases sampled were caused by this species. This has significant implications for diagnosis, treatment and control of malaria in this part of northwest Africa, so reporting and advice by the World Health Organization and other authorities need to reflect the epidemiological situation.
